# Effects of a staff education programme about person-centred care and promotion of thriving on relatives’ satisfaction with quality of care in nursing homes: a multi-centre, non-equivalent controlled before-after trial

**DOI:** 10.1186/s12877-020-01677-7

**Published:** 2020-08-01

**Authors:** Qarin Lood, Karin Sjögren, Ådel Bergland, Marie Lindkvist, Marit Kirkevold, Per-Olof Sandman, David Edvardsson

**Affiliations:** 1 The Medical Faculty, Department of nursing, Umeå University, Vårdvetarhuset, Hus A, plan 5, 90187 Umeå, Sweden; 2 Institute of Neuroscience and Physiology, Department of Health and Rehabilitation, Sahlgrenska Academy, Centre for Ageing and Health – AgeCap, University of Gothenburg, Box 455, 40530 Gothenburg, Sweden; 3 College of Science, Health and Engineering, School of Nursing and Midwifery, La Trobe University, Level 4 Austin Tower, PO Box 5555, Heidelberg, VIC 3084 Australia; 4 Lovisenberg Diaconal University College, Lovisenberggaten 15b, 0456 Oslo, Norway; 5 Department of Epidemiology and Global Health, Umeå University, Försörjningsvägen 7D, 907 37 Umeå, Sweden; 6 Faculty of Medicine, Institute of Health and Society, Oslo University, Nedre Ullevål 9, 0850 Oslo, Norway; 7 Institute of Nursing and health promotion, Oslo Metropolitan University, Pilestredet 32, 0130 Oslo, Norway; 8 NVS, Department of Nursing, Karolinska Institutet, Stockholm, Sweden

**Keywords:** Care home, Caring environment, Family members, Nursing, Older people, Person-centred care, Person-centered care, Residential aged care

## Abstract

**Background:**

As part of a nursing home intervention study, the aim of this paper was 1) to evaluate the effects of a staff education programme about person-centred care and promotion of thriving on relatives’ satisfaction with quality of care and their perceptions of the person-centredness of the environment, and 2) to outline factors of importance to explain the variance in relatives’ satisfaction with quality of care. Relatives are often referred to as vital for the operationalisation of person-centredness in nursing homes, representing an important source of information for care planning and quality of care assessments. However, the evidence for effects of person-centredness in nursing homes on relatives’ experiences is sparse and little is known on what could explain their satisfaction with the quality of care.

**Methods:**

A multi-centre, non-equivalent controlled group before-after design with study sites in Australia, Norway and Sweden. Staff in the intervention group participated in a 14-month education on person-centredness, person-centred care, thriving and caring environment. Staff in the control group received a one-hour lecture before the intervention period. Data were collected at baseline, after the intervention and six months after the end of the intervention, and analysed using descriptive statistics, a generalised linear model and hierarchical multiple regression.

**Results:**

In general, relatives from both the intervention and control nursing homes were satisfied with the quality of care, and no statistically significant overall between-group-effects of the intervention were revealed on satisfaction with quality of care or perceptions of the person-centredness of environment. A person-centred environment in terms of safety and hospitality were identified as factors of prominent importance for the relatives’ satisfaction with the quality of care.

**Conclusion:**

The findings of this paper provide a foundation for future research in terms of intervention design in nursing home contexts. Staff availability, approachability, competence and communication with relatives may be important factors to consider to improve quality of care from the perspective of relatives, but more research both with and for relatives to people living in nursing homes is necessary to identify the keys to success.

**Trial registration:**

ClinicalTrials.gov-NCT02714452. Registered on March 19, 2016.

## Background

Considering the increase of older people in need of nursing home care and the enduring nature of quality of nursing home care concerns, there is a need for more research on how to improve the quality of nursing home care. In a recent study, Lood et al. [1] suggest that a more person-centred environment, with emphasis on safety and hospitality, is related to higher quality of care as perceived by relatives [1]. In addition to this, older people living in nursing homes and their relatives seem to associate a good life at the nursing home with both physical and psychosocial aspects of the care environment [2]. This has also been described in the theoretical literature on person-centred care, referring to the importance of context where care is provided for implementation of person-centred care. Both the physical environment and the way care is organised are seen as essential for person-centred care, representing how people experience the environment, how they feel when they are there, and what people do in the environment [3]. This indicates that there might be positive relationships between quality of care, care environment and person-centred care, but there is little empirical evidence to support this hypothesis.

Following the introduction of person-centred care in nursing homes, there has been a gradual shift towards care models that aim to involve the person in need of care and their relatives in the care process [4, 5]. This shift is driven by the notion of people as persons who are more than their disease or disability, putting emphasis on experiences of illness rather than the disease itself. As described by Waters and Buchanan [6], person-centredness incorporates an ethcial approach to human services, grounded in the understanding of people as persons who are; capable of making decisions, in need of other people, and worthy of love, participation, engagement, and social inclusion [6]. Representing a unifying basis for a diversity of healthcare specialities and fields [7], person-centredness is increasingly being operationalised into person-centred care in geriatric and gerontological research and clinical practice [8], with the general aim to improve the quality of care [6]. Attending to the holistic needs of each person and striving for involvement of the person in need of care and their relatives in decision making processeses [3], person-centred care counter-balances deficit-focused care models [8]. This implies recognition, respect and trust for one another, and care staff are encouraged to see the world through the older person’s eyes, to be able to respond to their needs and build trustful relationships [8]. By involving both older people and their relatives in the care process, person-centred care allows staff to understand different life experiences and to take individual interests and social networks into account when planning the care [9–11]. Moreover, McCormack and McCance [3] describe the care environment as central for person-centred care, representing supportive organisational systems, effective relationships between staff, shared decision-making and sharing of power, as well as providing opportunities for risk-taking and innovation [3]. In nursing homes, the environment’s relation to person-centred care has been described in relation to homelike and personalised environments that allow people to acknowledge and recognise each other and what they enjoy doing [12]. Embodying a community where older people live together in a place where other people come to work, nursing homes need to be sensitive to both individual and collective needs, of both people who live and work there [13]. Consequently, quality of nursing home care ought to be assessed by several different quality indicators and from different perspectives [14]. In line with person-centredness, the perspective of the person in need of care is of utmost importance when assessing quality of care. However, the cognitive and physical status of the majority of people living in nursing homes may impede their ability to express their perceptions of the quality of care [15]. Relatives could then present an important source of information, representing the older person’s voice in assessments of nursing home care. Previous explorations of person-centred care in nursing homes from the perspective of relatives have, however, been mainly qualitative [16], and little is known on the associations between the level of person-centred care in nursing homes and relatives’ satisfaction with the quality of care.

Relatives to older people living in nursing homes often take on the responsibility for monitoring the quality of care and for conveying the older person’s desires and needs for support [17]. As described by Björnsdòttir et al. [18], frail older people may rely on their relatives for assistance [18], and when an older person needs to move into a nursing home, relatives reportedly may experience feelings of sadness, guilt, loss of control and disempowerment [19, 20]. Such feelings could be relieved by involvement of relatives in the care process, which optimally could facilitate a sense of purpose and opportunity to contribute to the development of support relevant for the older person [21]. Relatives’ involvement in nursing home care has also been described as an important aspect of quality of care from the perspective of both the person living in the nursing home and the relative [2], and relatives have been highlighted as important resources to provide different experiences of nursing home services [22]. However, relatives seem to experience difficulties with involvement in the care process and may perceive the quality of care as low because of this, even when they are satisfied with the direct care provided to the older person [1].

Efforts have been made to implement and evaluate person-centred care in nursing homes, but the evidence for effects of person-centred care on relatives’ perceptions of the quality of care is sparse. As described by Bauer et al. [23], there has been a predominant focus on different aspects of involving relatives in the care process [24], on relatives’ relationships with staff [23], and on ethical aspects of care [2, 25, 26]. To the best of our knowledge, no previous studies have reported effects of person-centred care interventions in nursing homes on relatives’ satisfaction with the quality of care. Therefore, the U-Age Nursing Home study [27] was developed to explore effects and meanings of a staff education programme about person-centred care and promotion of thriving in nursing homes, from the perspective of the people living there, their relatives, and nursing home staff [27]. This paper puts focus on the perspective of relatives, exploring how they perceived the quality of care and person-centredness of the environment after staff had participated in the education.

## Methods

As part of the U-Age Nursing Home study [27], the aim of this paper was twofold; 1) to evaluate the effects of a staff education programme about person-centred care and promotion of thriving on relatives’ satisfaction with the quality of care and their perceptions of the person-centredness of the environment, and 2) to outline factors of importance to explain the variance in relatives’ satisfaction with quality of care. The U-Age Nursing Home study had a multi-centre, non-equivalent controlled group before-after design [27], with three international study sites in Australia, Norway and Sweden. Each site had one intervention nursing home and one control nursing home, included in the study based on the following inclusion criteria: 1) managers expressing a need for improvements with regards to person-centred care, 2) managers expressing a willingness to participate and facilitate the study, 3) consisting of at least 50 beds, and 4) having at least 50 staff members. The inclusion of nursing homes to intervention or control was purposively conducted at each study site using the research group’s clinical networks.

### Intervention

Direct care staff at the intervention nursing homes participated in a researcher-moderated education programme on the theoretical understanding of person-centredness, person-centred care, thriving and caring environment over a 14-month period. The intervention was built on an interactive pedagogical framework [27], based on the view that all participants could contribute to new knowledge and development through their experience and competence. This involved a process where knowledge from theory and philosophy was integrated with practice through theoretical workshops and practice-based activities. First, researchers presented the best available evidence for person-centredness and person-centred care to promote thriving and a caring environment in nursing homes. Second, the evidence was discussed with participating staff, with regards to how to understand and implement current knowledge into daily practice. Third, means for site-based reflection and evaluation activities were identified and planned for with participating staff, and finally, staff participated in follow-up discussions and analyses of the site-based reflections and evaluation activities. This process was repeated for three dimensions, representing an operationalisation of person-centredness, person-centred care, thriving and caring environment based on the research group’s previous studies. These dimensions were: 1) *Doing a little extra*, encompassing the willingness to do something that the other person did not expect, with the aim to facilitate thriving and happiness. Converting a willingness to serve into an actual doing of something extra for someone living in the nursing home, for their relative or for a colleague, doing a little extra represented a way for staff to both identify and do something that really mattered for another person. 2) *Developing a caring environment*, which put focus on how a physical and psychosocial caring environment could promote thriving among people living in the nursing home and their relatives, and among staff. This dimension encompassed observations of the environment, and dialogues with the people living in the nursing home as well as with their relatives, with the aim to identify needs for improvement in the nursing home environment. These identified needs were then prioritised and site-specific changes to the environment were carried out based on the observations and dialogues. 3) *Assessing and meeting highly prioritised psychosocial needs* was the third and final dimension, representing collaboration between the participating staff, the people living in the nursing home and their relatives. Focus in the collaboration was dialogues to identify the older persons’ psychosocial needs, such as needs for communion or intellectual stimulation [27].

### Control

Direct care staff at the control nursing homes participated in one researcher-led lecture on the theoretical understanding of person-centredness, person-centred care, thriving and caring environment. The lecture was given after the baseline data collection, and no further contact between control group and researchers was made until after the intervention when follow-up data were collected.

### Setting and participants

The Australian and Swedish nursing homes were situated in small rural towns with approximately 1500 inhabitants each, and the Norwegian nursing homes were situated in a larger city. The size of the nursing home ranged from 50 to 127 beds, and the the older persons’ length of stay had a median of 24 months (1–360 months). Care staff were available 24 h for the people living in the nursing homes, and allied health professionals were available for care and rehabilitation when needed. A majority (90%) of the staff who participated in the intervention were women, with a mean age of about 43 years. They were enrolled nurses (61%), registered nurses (22%), care assistants (12%) and allied health professionals (5%) with a mean work experience of 13 years in aged care.

One relative per person living in each of the participating nursing homes was invited to participate in the study by completing a study specific survey. Eligibility criteria for participation were 1) being a relative to a person who had lived at the nursing home for at least one month at the time of data collection, 2) visiting the nursing home at least once a month, and 3) being able to complete the study survey, which was formulated in English, Norwegian or Swedish depending on the study site. A broad definition of relatives was applied, including people who the person living in the nursing homes had registered as their contact person.

**Fig. 1:**
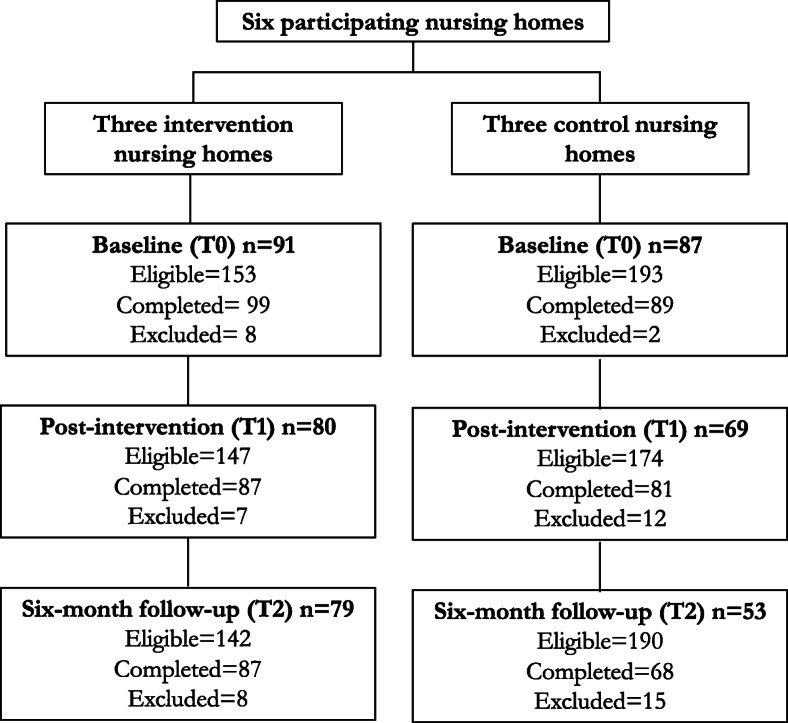
Flowchart over inclusion of participants

At baseline, 346 surveys were sent out and 188 (54%) were returned. After inspection of the data 10 surveys were excluded because the respondent did not fulfil the eligibility criteria or because there were missing data on all or most of the background characteristics. At the first post-intervention follow-up, 321 surveys were sent out, 168 (52%) were returned and 19 were excluded because the respondent did not fulfil the eligibility criteria or because there were missing data on all or most of the background characteristics. At the second post-intervention follow-up, 322 surveys were sent out and 155 (48%) were returned. Twelve surveys were excluded because they did not fulfil the eligibility criteria or because they had not entered any information on background characteristics, and 11 were excluded because they were completed after a grass fire that resulted in all people living at the control facility in Australia being evacuated. The evacuation might have coloured the relatives’ perceptions, making it difficult to compare their answers to the rest of the sample. Consequently, data from 459 surveys were included in the study; 178 at baseline (91 intervention, 87 control), 149 at the first follow up (80 intervention, 69 control), and 132 at the second follow up (79 intervention, 53 control). For more information on the inclusion of participants, see Fig. 1.

### Data collection

Data were collected at baseline (T0, March to June 2016), after the intervention (T1, September to November 2017), and again at six months after the end of the intervention (T2, March to May 2018). Since mortality rates among people who live in nursing homes tend to be high [28], relatives were invited at each data collection time point to avoid high drop-out rates. All eligible relatives at each data collection time point were informed by the nursing home management about the aims of the study and that they would be contacted by mail and asked to complete a study survey. Before completing the surveys, all relatives received information on the study aim and conduct, including information on the voluntary nature of participation, that no personally identifiable data on themselves or their relatives would be collected, that they would remain completely anonymous throughout the study, including the reports, and that they were free to decline or withdraw their participation at any stage without risking negative effects for themselves or their relative. The survey sent to relatives comprised questions on their satisfaction with the quality of care (primary outcome) and perceived person-centredness of the environment (secondary outcome). To provide data on contextual aspects, the survey also comprised questions on the relatives’ background characteristics, i.e. age in years, sex (1 = woman, 2 = man), relation to the person living in the nursing home (1 = child, 2 = partner/other), frequency of visits (1 = every week, 2 = every month), and length of stay in months.

#### Satisfaction with the quality of care

Data on the primary outcome satisfaction with the quality of care were generated by the Pyramid questionnaire, a validated tool developed to collect information on satisfaction with the quality of care from the perspective of people in need of care and their relatives [29]. The tool consists of 31 questions on eight quality of care indices: Information, nursing staff, caring processes, activity, contact, social support, participation, and work environment. Examples of questions are: “Is your relative well cared for regarding meals/physical transfers/physical training or physiotherapy?” and “Do you participate in the planning of your relative’s care?”. Each question has responses on a four-point Likert-type scale, from “1=No not at all” to 4 = Yes to a great degree”. Ten questions also had the response “Not applicable”, these responses were treated as missing values during the analyses. Sum scores for the entire tool as well as for sub-scales were calculated for each relative. There are two negatively worded questions in the tool (“Do you think that staff work under stress?” and “Do you think that staff have a heavy workload?”). These were reversed for analytical purposes. There was also an overall rating of the quality of care on a visual analogue scale (VAS), ranging from “1 = Very negative” to “10 = Very positive”. Reliability for the sum score in this sample was tested by Cronbach alpha, showing good reliability at baseline (α = 0.95), first (α = 0.95), and second (α = 0.93) follow up.

#### Person-centredness of the environment

The secondary outcome perceived person-centredness of the environment was measured using the Person-centred Climate Questionnaire - Family version (PCQ-F) [30]. The tool consists of a total of 17 items, divided into three subscales; safety, everydayness, and hospitality. Examples of questions are; “I experience this facility as a place where my relative receives the best possible care” (safety subscale), “I experience this facility as a place where people talk about everyday life and not just illness” (everydayness subscale), and “I experience this facility as a place where staff make extra efforts for my relative’s comfort” (hospitality subscale). Responses are rated on a six-point Likert-type scale, from ‘0 = No, I disagree completely’, to ‘5 = Yes, I agree completely’. To summarise the scale, a total sum score is calculated, ranging from 0 to 85, and a higher score indicates a higher degree of person-centredness. The psychometric properties of the PCQ-F have been previously tested, showing high content validity and internal consistency [30]. Reliability in this sample was tested by Cronbach alpha, showing good reliability at baseline (α = 0.95), first (α = 0.95), and second (α = 0.95) follow up.

### Sample size calculation

In order to achieve 85% power (alpha = 0.05, two-tailed) for detecting a pre-post group mean difference of at least medium effects size, the number of relatives in the intervention and control group needed to be at least 150 in each group.

### Data analyses

Data were entered into the Statistical Package for the Social Sciences (SPSS) version 23.0 (IBM Corp., Armonk, NY, 2013, and analysed using descriptive and analytical methods. Before analysis, data were checked for outliers, normality, linearity, homoscedasticity, multicollinearity, and common method bias [31].

Frequencies, medians, ranges, means and standard deviations (SD) were computed to describe the participants’ background characteristics and to give a descriptive understanding of the primary outcome satisfaction with the quality of care. Between group differences at baseline were tested using independent samples t-test, Mann-Whitney U-test, or Chi-square for independence.

The effects of the intervention were analysed using a generalised linear model, adjusting for study site. First, the impact of the intervention on the relatives’ satisfaction with the quality of care (sum score and overall score) was analysed, including time for data collection (T0, T1, T2), allocation (intervention, control) and study site (Australia, Norway, Sweden). To investigate changes between the groups over time, the cross-term time for data collection*allocation was included in the model. A two-tailed alpha of < 0.05 was considered to indicate statistical significance and partial eta^2^ of > 0.05 were regarded as clinically significant effect sizes. Second, the same model was applied to test the impact of the intervention on the relatives’ perceptions of the person-centredness of the environment (total score, and sub-scales).

To outline factors of importance to explain the variance in relatives’ satisfaction with quality of care, associations between person-centredness of the environment and satisfaction with the quality of care at each of the two follow-up data collections were explored by hierarchical multiple regression models with data from relatives in both intervention and control group. The models controlled for the relatives’ background characteristics, study site and number of people living at the nursing home. Satisfaction with the quality of care was entered as the dependent variable, and independent variables were entered in two steps: 1) relatives’ age, sex, relation to the person living in the nursing home, and frequency of visits, the older person’s length of stay, study site and number of people living at the nursing home, and 2) person-centred climate subscales (safety, everydayness, hospitality). Standardised beta was used for comparisons of the significance of the associations across the explanatory variables, and model fit was assessed by R^2^ and a two-tailed probability value of < 0.05 was considered to indicate statistical significance.

Imputation was applied when less than 10% of the answers of The Pyramid questionnaire and the PCQ-F were missing. For those participants, the missing values were replaced with the participant’s mean value for the scale [32].

## Results

The total sample of relatives had a mean age of 64.7 years (SD = 12.5) and the majority were women (64%), children to the person living in the nursing home (68%), and visited the nursing home every week (68%). There were statistically significant differences with regard to age and number of relatives in the intervention and control groups at each study site at baseline (T0), and with regard to number of relatives in the intervention and control groups at each study site at the second follow up (T2). No other statistically significant between group differences were detected. For more information on the background characteristics of the relatives, please see Table 1.

**Table 1:** Relatives’ background characteristics at baseline (T0), first (T1) and second (T2) follow-up

Background characteristics	Total sample (***n*** = 459)	T0			T1			T2		
		Intervention (***n*** = 91)	Control (***n*** = 87)	p	Intervention (***n*** = 80)	Control (***n*** = 69)	p	Intervention (***n*** = 79)	Control (***n*** = 53)	p
Age in years, mean (SD)	64.7 (12.5)	61.6 (12.3)	67.1 (14.0)	**< 0.01**	64.1 (11.4)	66.3 (12.5)	0.26	64.0 (10.8)	65.3 (13.2)	0.46
Sex, n (%)										
Female	293 (64.4)	63 (70)	49 (56.3)	0.06	56 (71.8)	40 (58.8)	0.14	52 (65.8)	33 (62.3)	0.82
Male	162 (35.6)	27 (30.0)	38 (43.7)		22 (28.2)	28 (41.2)		27 (34.2)	20 (37.7)	
Relation to person living in the nursing home, n (%)										
Son/daughter	309 (67.6)	67 (73.6)	52 (59.8)	0.15	54 (67.5)	45 (65.2)	0.92	57 (73.1)	34 (65.4)	0.33
Partner/other	148 (32.4)	24 (26.4)	35 (40.2)		26 (32.5)	24 (34.8)		21 (26.9)	18 (34.6)	
Frequency of visits, n (%)										
Every week	322 (67.6)	63 (70.0)	60 (69.0)	1.00	55 (68.8)	49 (72.1)	0.80	58 (73.4)	37 (69.8)	0.80
Every month	135 (29.5)	27 (30.0)	27 (31.0)		25 (31.2)	19 (27.9)		21 (26.6)	16 (30.2)	
Older person’s length of stay in months, median (range)	24 (1–360)	24.0 (2–360)	24.0 (1–108)	0.93	27.5 (2–240)	24 (2–136)	0.41	24.0 (2–144)	23 (3–144)	0.62
Study site, n (%)										
Australia	131 (28.5)	30 (33.0)	18 (20.7)	**< 0.05**	32 (40.0)	18 (26.1)	0.20	33 (41.8)	0 (0.0)	**< 0.01**
Norway	148 (32.2)	35 (38.5)	28 (32.2)		19 (23.8)	20 (29.0)		23 (29.1)	23 (43.4)	
Sweden	180 (39.2)	26 (28.5)	41 (47.1)		29 (36.3)	31 (44.9)		23 (29.1)	30 (56.6)	

T0 = Baseline, T1 = First follow up, T2 = Second follow up

Descriptive analyses of the data from the intervention group showed a decrease in the overall satisfaction with the quality of care from baseline to the first follow up, and an increase from the first to the second follow up. In the control group, the relatives’ ratings also decreased from baseline to the first follow-up, for a slight recover at the second follow-up. Both intervention and control group were most satisfied with the quality of social support and caring processes, and least satisfied with the quality of information and activity. There were a few statistically significant differences between the relatives’ ratings in the intervention and control group. At baseline, the relatives in the control group were more satisfied with the quality of caring processes than the intervention group (*p* = 0.01). At the second follow-up the control group were more satisfied with the quality of information (*p* = 0.02) and nursing staff (*p* = 0.03) than the intervention group, whereas the relatives in the intervention group were more satisfied with the quality of the work environment (*p* = 0.03). Considering the person-centredness of environment, the relatives in both the intervention and control group perceived the environment as more person-centred in terms of safety than in terms of everydayness and hospitality. There was a statistically significant difference between the intervention group and control group at the first follow-up with relatives in the intervention group perceiving the environment as more person-centred in terms of everydayness (*p* = 0.03). For more information on the descriptive analyses, please see Table 2.

**Table 2:** Descriptive statistics for quality of care and person-centredness of environment, including subscales

	Intervention			Control			p		
Variable	T0	T1	T2	T0	T1	T2	T0	T1	T2
Satisfaction with the quality of care, sum score mean (SD), (range 0–124)	95.81 (13.74)	90.01 (11.93)	95.81 (11.70)	95.55 (14.19)	91.51 (14.48)	94.15 (13.59)	0.96	0.34	0.36
Information, item mean (SD)	2.52 (0.95)	2.41 (0.84)	2.48 (1.01)	2.89 (0.82)	2.63 (0.97)	2.80 (0.80)	0.45	0.19	**0.02**
Nursing staff, item mean (SD)	3.34 (0.54)	3.25 (0.52)	3.03 (0.48)	3.24 (0.59)	3.24 (0.66)	3.13 (0.64)	0.18	0.14	**0.03**
Caring processes, item mean (SD)	3.45 (0.60)	3.52 (0.48)	3.56 (0.45)	3.61 (0.48)	3.46 (0.58)	3.41 (0.53)	**0.01**	0.21	0.21
Activity, item mean (SD)	2.60 (0.85)	2.44 (0.83)	2.41 (0.86)	2.64 (0.89)	2.71 (0.96)	2.70 (0.84)	0.64	0.14	0.46
Contact, item mean (SD)	3.42 (0.57)	3.35 (0.60)	3.41 (0.56)	3.43 (0.55)	3.41 (0.57)	3.31 (0.59)	0.38	0.86	0.87
Social support, item mean (SD)	3.47 (0.47)	3.37 (0.56)	3.44 (0.46)	3.42 (0.57)	3.37 (0.64)	3.19 (0.63)	0.20	0.49	0.05
Participation, item mean (SD)	2.79 (0.83)	2.72 (0.81)	2.84 (0.81)	2.85 (0.77)	2.71 (0.84)	2.73 (0.79)	0.54	0.62	0.69
Work environment, item mean (SD)	3.11 (0.44)	3.06 (0.46)	3.11 (0.44)	3.10 (0.44)	3.05 (0.50)	2.99 (0.34)	0.58	0.64	**0.03**
Person-centredness of the environment, sum score mean (SD) (range 0–85)	68.73 (11.10)	66.51 (10.91)	68.14 (10.80)	65.14 (13.67)	63.62 (16.00)	62.76 (13.74)	0.17	0.06	0.16
Safety, item mean (SD)	4.21 (0.62)	4.04 (0.67)	4.12 (0.62)	4.11 (0.81)	3.94 (0.90)	3.94 (0.80)	0.13	0.21	0.15
Everydayness, item mean (SD)	3.83 (0.87)	3.73 (0.87)	3.83 (0.80)	3.38 (1.03)	3.47 (1.12)	3.36 (0.99)	0.23	**0.03**	0.10
Hospitality, item mean (SD)	3.74 (0.92)	3.66 (0.88)	3.79 (0.95)	3.50 (1.06)	3.43 (1.23)	2.27 (1.02)	0.24	0.05	0.38

### Effects of the intervention

The general linear model did not identify any statistically significant impacts of the intervention on relatives’ satisfaction with the quality of care or their perceptions of the person-centredness of the environment. For more information on the results of the analysis, see Table 3.

**Table 3:** Baseline and follow up data for relatives related to primary and secondary outcomes after controlling for study site (Australia, Norway and Sweden)

	Intervention group Mean (SE)			Control group Mean (SE)			Effects	
Measures	T0	T1	T2	T0	T1	T2	Change between groups from T0 to T1	Change between groups from T0 to T2
Satisfaction with the quality of care, sum score (0–124)^a^	96.7 (2.2)	93.0 (2.3)	97.1 (2.5)	100.8 (2.0)	97.9 (2.4)	94.9 (2.5)	*P* = 0.86 mean change (SE) = 0.76 (4.41) partial eta^2^ = 0.000	*p* = 0.16 mean change (SE) = −6.29 (4.51) partial eta^2^ = 0.009
Satisfaction with the quality of care, VAS [1–10]	8.0 (0.2)	7.9 (0.2)	8.2 (0.2)	7.9 (0.2)	7.7 (0.2)	7.7 (0.2)	*P* = 0.67 mean change (SE) = − 0.16 (0.37) partial eta^2 =^ 0.000	*P* = 0.30 mean change (SE) = − 0.41 (0.39) partial eta^2^ 0.003
Person centredness of the environment, sum score (0–85)^b^	68.7 (1.3)	66.4 (1.5)	67.8 (1.4)	65.8 (1.4)	64.1 (1.5)	64.3 (1.8)	*p* = 0.86 mean change (SE) = 0.52 (2.84) partial eta^2^ = 0.000	*p* = 0.54 mean change (SE) = −0.59 (2.99) partial eta^2^ = 0.000

T0 = Baseline, T1 = First follow-up, T2 = Second follow-up

^a^Higher scores indicate higher satisfaction

^b^Higher scores indicate a more person-centred environment

### Factors explaining the variance in relatives’ satisfaction with the quality of care

At the first follow up, the hierarchical multiple regression model shown in Table 4 revealed that the safety sub-scale of PCQ-F was the only explanatory variable that had a statistically significant association with the relatives’ satisfaction with the quality of care (*p* < 0.01), controlling for background characteristics, study site and number of residents at the nursing home. This means that relatives who perceived the environment as more person-centred in terms of safety also were more satisfied with the quality of care (β = 0.67). The model was statistically significant and explained 75% (adjusted R^2^ 0.75) of the variance (F change (3, 60) = 52.75, *p* < 0.01). No problems with multicollienarity were detected based on tolerance values ranging from 0.310 to 0.941 and VIF values ranging from 1.062 to 3.267. The association between safety and satisfaction with the quality of care remained at the second follow up (Table 4). The hierarchical multiple regression model conducted on data from the second follow-up showed that safety had the strongest unique association with the relatives’ satisfaction with the quality of care (β =0.71, *p* < 0.01). Hospitality also had a strong and statistically significant association with the relatives’ satisfaction with the quality of care (β =0.32, *p* = 0.01). The model was statistically significant (F change (3, 50) = 62.26, *p* < 0.01), and explained 77% (adjusted R^2^ 0.77) of the variance in satisfaction with the quality of care. No problems with multicollinearity were detected based on tolerance values ranging from 0.333 to 0.949 and VIF values ranging from 1.054 to 2.999 (Table 4).

**Table 4:** Satisfaction with the quality of care by selected explanatory variables at the first and second follow up (pooled data from the intervention and control group)

	First follow-up (T1)							Second follow-up (T2)								
	Step 1				Step 2				Step 1				Step 2			
	β	t	p	Part corr.	β	t	p	Part corr.	β	t	p	Part corr.	β	t	p	Part corr.
(Constant)		5.78	**< 0.01**			3.24	**< 0.01**			5.93	**< 0.01**			3.50	**< 0.01**	
Age	0.08	0.61	0.55	0.07	0.05	0.70	0.49	0.04	0.07	0.50	0.62	0.07	0.02	0.31	0.76	0.02
Sex	0.24	2.06	**0.04**	0.23	0.12	1.89	0.07	0.11	0.24	1.75	0.09	0.23	0.08	1.29	0.20	0.08
Relation to the resident	0.16	1.25	0.22	0.14	−0.02	−0.29	0.78	−0.02	−0.08	−0.51	0.61	−0.07	−0.13	−1.79	0.08	−0.11
Frequency of visits	0.16	1.36	0.18	0.15	0.01	0.16	0.87	0.01	−0.11	−0.82	0.42	−0.11	−0.08	−1.22	0.23	−0.08
Resident’s length of stay (months)	−0.29	−2.48	**0.02**	−0.28	−0.12	−1.73	0.09	−0.10	−0.02	−0.18	0.86	−0.02	0.09	1.43	0.16	0.09
Study site	−0.23	−1.86	0.07	−0.21	−0.02	−0.34	0.74	−0.02	0.01	0.10	0.92	0.01	0.11	1.66	0.10	0.10
Number of resident beds	0.03	0.24	0.81	0.03	−0.00	−0.05	0.96	−0.00	0.13	0.97	0.34	0.13	0.04	0.57	0.58	0.04
PCQ safety					0.67	6.15	**< 0.01**	0.37					0.71	6.63	**< 0.01**	0.41
PCQ everydayness					−0.00	−0.05	0.96	−0.00					−0.12	−1.29	0.21	−0.08
PCQ hospitality					0.19	1.78	0.08	0.11					0.32	2.97	**0.01**	0.18

*β* Standardised beta coefficients

## Discussion

The aim of this paper was 1) to evaluate the effects of a staff education programme about person-centred care and promotion of thriving on relatives’ satisfaction with quality of care and their perceptions of the person-centredness of the environment, and 2) to outline factors of importance to explain the variance in relatives’ satisfaction with quality of care. Although the analyses demonstrated no statistically significant overall effects of the staff education programme on either of the outcomes stated above, a person-centred environment in terms of safety was identified as a factor of prominent importance for the relatives’ satisfaction with the quality of care. As the aspect with the strongest association with the relatives’ satisfaction with the quality of care, safety remained statistically significant across three separate data collections with partially different relatives. Interpreted in relation to the publication by Edvardsson, Sandman and Rasmussen [33], this indicates that the relatives felt safe that their loved one received the best possible care, that staff responded quickly when they needed help, and that staff were knowledgeable and easily approachable [33]. These factors seem to be of very high importance to the satisfaction with quality of care as the model explained 75% of the variance in ratings. Statistically significant associations were also detected between a hospitable environment and satisfaction with the quality of care, indicating that relatives associated quality of care with a welcoming feeling, and an environment where care exceeded their expectations [33]. In the light of these analyses, it may seem surprising that the staff education programme on person-centred care did not have an effect on the relatives’ satisfaction with care. The education programme involved activities to integrate theoretical knowledge on person-centred care into daily practice, but the findings of this study indicate that this may not have led to an actual change to the care provided, at least not as perceived by the relatives. Preliminary findings that will be reported in a separate paper, indicate that the staff education programme may have had an effect on the older people’s thriving. However, the design of the larger intervention study did not involve explorations of the implementation of knowledge to daily practice. In hindsight, the authors acknowledge that studying the implementation process could have provided a greater understanding of the lack of overall effects from the perspective of relatives. A suggestion for future research is therefore to include both quantitative and qualitative evaluations of interventions, to study implementation of knowledge and actual behaviour change among staff. Moreover, the findings presented in this paper also suggest that special attention should be paid to safety and hospitality aspects of the care environment in order to improve relatives’ satisfaction of the quality of care. Moreover, even if the education encompassed information on how, when and why relatives should be involved in the care process, many of the participating relatives only visited the nursing home once a month, and perhaps only for a couple of hours at a time. As a consequence, some of the relatives may not have been made aware of the changes occurring at the intervention nursing homes. Another suggestion for future research is therefore to design interventions that specifically aim to involve relatives in the care process and improve the communication between relatives and staff. This has also been described in a qualitive study by Jakobsen and Sellevold et al. [34], exploring relatives’ experiences with quality of nursing home care. With focus on expectations, they describe that although relatives may be at an overall level satisfied with staff engagement and inclusion, feelings of powerlessness, guilt and distrust can be common when not being able to understand what is happening and not feeling competent enough to make medical decisions themselves [34]. A focus for future studies could thus be explorations on how to invite relatives to people living in nursing homes into the decision-making process, and how to improve trust in staff competence and willingness to act upon relatives’ expectations. As described by Wiig et al. [35] establishing quality and safety in healthcare may also require interaction and collaboration across different stakeholders [35], and person-centred care is suggested as a way to put focus on human interaction in healthcare settings [16]. Nevertheless, few studies exist that evaluate the effects of person-centred care on the quality of human interactions. Chenoweth et al. [36] report findings from one of few such studies, documenting positive effects of person-centred care on the interaction between older people living in nursing homes and nursing home staff, as well as on the older persons’ emotional responses to care. What they did not do in that study was to evaluate the effects on relatives’ experiences [36], which is why they decided to do a second study to explore the intervention qualitatively, in order to understand whether or not person-centred care in nursing homes could make a difference to relatives’ experiences of the care. Their qualitative study uncovered the importance of person-centred care for relatives in terms of experiences of staff responsiveness and involvement of relatives in care related decisions. They did, however, also reveal difficulties for staff to explain what it is they do when working in a person-centred way, and revealed existing difficulties for relatives to be involved in the care process even if they visited the nursing home on a regular basis [16]. This and the above studies indicate a need for further research into translation and communication of the ethical foundations for person-centred care to relatives of people living in nursing homes, and how to provide opportunities for relatives to be involved in a way perceived as meaningful and relevant for the quality of care.

The findings also showed a relative stability in the intervention group’s satisfaction with the quality of care and perceptions of the person-centredness of the environment as compared to the control group which had a slight decrease from baseline to both first and second follow-up. Although there were no statistically significant overall differences when comparing the groups, it is possible that the intervention had effects which the instruments chosen to measure the study outcomes were unable to detect. Considered in the light of previous research findings by Chenoweth et al. [16] the findings suggest that there is also a need for qualitative evaluations of intervention studies in nursing homes. Even though the findings provide evidence for strong associations between person-centredness of the environment and relatives’ satisfaction with the quality of care, there could be other factors, not included in this study, that may have an influence on relatives’ experiences of person-centred care in nursing homes. Alongside evaluations of the implementation of interventions from the perspective of staff, it is likely that qualitative explorations of relatives’ experiences of nursing home interventions could increase the understanding of what really matters to them and the people they care for. Moreover, even if this paper did not reveal any statistically significant differences across Australia, Norway and Sweden, it is possible that relatives’ perceptions could differ across contexts and cultures. This calls for further international explorations across different nursing home contexts, to reach a deeper understanding of what really matters to relatives and the people they care for. Moreover, a greater focus on involvement of relatives in the U-Age Nursing Home study could have improved the possibilities to influence their perceptions of the nursing home care and environment. Another suggestion for future intervention studies is therefore to involve all different nursing home stakeholders early in the research process to make sure that they are involved to the extent that they wish and that study outcomes are relevant to them. Such early and strong involvement of relatives in the designs of this study was not conducted, which is a limitation.

In an attempt to translate research-based knowledge into practice, the education programme developed for the U-Age Nursing Home study evaluated in this paper shared some key characteristics with other attempts to implement person-centred care in nursing homes. For instance, the PerCEN trial [36], that focused on the psychosocial needs of the people living in the nursing home, person-centred care planning and interaction, showed improvements in care interaction quality [36]. The Eden alternative [37] was another study designed to encourage involvement of relatives in nursing homes and to make the environment more home like, which was successful in terms of family satisfaction regarding care, staff skills, quality of activities, the environment, contentment of the people living in the nursing home, and relatives’ relationships with administration staff [37]. Previous research has also highlighted contextual factors, such as organisational culture, laws and regulations, as well as funding structures and external policies and incentives as explanations for variability in efforts to improve the quality of care in healthcare settings [38]. Thus, although the inclusion of nursing homes from three different countries could increase the generalisability of the findings, there could be contextual differences that may have led to suboptimal conditions for aggregating data for the intervention outcome analyses. More research is needed in order to optimise initiatives to implement person-centredness in nursing home settings across the globe, making it part of day-to-day practice in all nursing homes.

### Limitations

Intervention research in nursing homes is a challenging endeavour and the findings of this study need to be interpreted in the light of some limitations. First, data were collected from partly different groups of relatives at baseline and post-intervention follow-ups and no individual comparisons could be made. Although it was deemed the only viable option for conducting this study to include participants at point of data collection, this made it impossible to control for the threat of differential selection, which makes generalisation beyond the participants in the study difficult. Contextual information has been included in this paper for increased generalisability/transferability, and the inclusion of participants from three different countries, and both rural and urban settings reduces the risk of influence from changes to healthcare policy or nursing home organisation.

Second, from a person-centred perspective it seems reasonable that measuring the older persons’ own satisfaction with care would have been ideal. However, as the literature on person-centredness also suggest, both the person in need of care and their relatives are key players in the care process [4, 5]. In line with the study protocol [27], data on the people living in the nursing homes will therefore be reported in another publication from the research group.

Third, difficulties with recruiting relatives led to a response rate of about 50%. This led to the study being underpowered for the outcomes under study, which is an additional strong explanation to the inability to detect statistically significant overall effects. The descriptive analyses of the data did, however, not indicate trends of improvement in the intervention group, suggesting either that the intervention had no effect on relatives’ satisfaction with the quality of care, or that the Pyramid questionnaire was not sensitive enough to detect changes. The scientific literature on relatives’ satisfaction with the quality of care in nursing homes is sparse, which makes it difficult to compare the findings from this study with previous research. Finally, although providing international evidence for the relationship between person-centredness and relatives’ satisfaction with the quality of care, it is important to take into consideration the influence of federal and state oversight bodies may have on the quality of care, aspects that were not possible to influence by this intervention study.

## Conclusions

Although the findings of this study did not reveal any statistically significant overall effects of the intervention, they visualise aspects of care that may need specific attention in quality improvements programmes in nursing homes. In general, the relatives seemed satisfied with the quality of care processes, their contact with staff, and the social support they received. This pattern was prevalent in both intervention and control group, as well as in the total sample of relatives. Another important finding is the visualisation of the importance of a person-centred environment of safety, with focus on staff availability, approachability, competence, and communication with relatives, for relatives’ satisfaction with the quality of care in nursing homes. This gives an indication on what factors may be important to consider when designing interventions with the aim to improve nursing home care, but more research involving relatives is necessary in order to identify the keys to success with regards to operationalisation and implementation of person-centredness in nursing homes.

## Data Availability

The databases generated and/or analysed during the study are available from the corresponding author on reasonable request.

## Abbreviations

PCQ-F: Person-centred Climate Questionnaire - Family version
SD: Standard deviation
VAS: Visual analogue scale

## References

[1] Lood Q, Kirkevold M, Sjögren K, Bergland Å, Sandman P, Edvardsson D (2019). Associations between person-centred climate and perceived quality of care in nursing homes: a cross-sectional study of relatives’ experiences. J Adv Nurs.

[2] Bollig G, Gjengedal E, Rosland J (2016). Nothing to complain about? Residents’ and relatives’ views on a “good life” and ethical challenges in nursing homes. Nurs Ethics.

[3] McCormack B, McCance T (2010). Person-centred nursing: theory and practice.

[4] Koren M (2010). Person-centered care for nursing home residents: the culture-change movement. Health Aff.

[5] Grabowski D, O'Malley A, Afendulis C, Caudry D, Elliot A, Zimmerman S (2014). Culture change and nursing home quality of care. The Gerontologist.

[6] Waters R, Buchanan A (2017). An exploration of person-centred concepts in human services: a thematic analysis of the literature. Health Pol.

[7] McCormack B, Karlsson B, Dewing J, Lerdal A (2010). Exploring person-centredness: a qualitative meta-synthesis of four studies. Scand J Caring Sci.

[8] Edvardsson D, Winblad B, Sandman P (2008). Person-centred care for people with severe Alzheimer’s disease – current status and ways forward. Lancet Neurology.

[9] Curry A, Stark S (2000). Quality of service in nursing homes. Health Serv Manag Res.

[10] Kitwood T (1997). Dementia reconsidered: the person comes first.

[11] McCormack B (2003). A conceptual framework for person-centred practice with older people. Int J Nurs Pract.

[12] Edvardsson D, Fetherstonhaugh D, Nay R (2010). Promoting a continuation of self and normality: person-centred care as described by people with dementia, their family members and aged care staff. J Clin Nurs.

[13] Brown WC (2009). Developing community in care homes through a relationship-centred approach. Health Social Care Community.

[14] Castle N, Ferguson J (2010). What is nursing home quality and how is it measured?. The Gerontologist.

[15] Forbes D, Neufeld A (1997). Strategies to address the methodological challenges of client-satisfaction research in home care. Can J Nurs Res.

[16] Chenoweth L, Jeon Y-H, Stein-Parbury J, Forbes I, Fleming R, Cook J (2015). PerCEN trial participant perspectives on the implementation and outcomes of person-centered dementia care and environments. Int Psychogeriatr.

[17] Graneheim U, Johansson A, Lindgren B-M (2013). Family caregivers' experiences of relinquishing the care of a person with dementia to a nursing home: insights from a meta-etnographic study. Scand J Caring Sci.

[18] Björnsdòttir K, Ceci C, Purkis M (2013). The ‘right’ place to care for older people: home or institution?. Nurs Inq.

[19] Logue R (2003). Maintaining family connectedness in long-term care. J Gerontol Nurs.

[20] Swann J (2006). The journey into care. Nurs Residential Care.

[21] Kellet U (2007). Seizing possibilities for positive family caregiving in nursing homes. J Clin Nurs.

[22] Rognstad M, Sagbakken M, Nåden D (2015). Family members’ role as resources and collaborating partners: a study focusing on dementia and long term stay in a nursing home. Nordic J Nursing Res.

[23] Bauer M, Fetherstonhaugh D, Tarzia I, Chenco C (2014). Staff-family relationships in residential aged care facilities: the views of residents’ family members and care staff. J Appl Gerontol.

[24] Lopez R, Mazor K, Mitchell S, Givens J (2013). What is family-centered care for nursing home residents with advanced dementia. Am J Alzheimers Dis Other Dement.

[25] Heggestad A, Nortvedt P, Slettebø A (2015). Dignity and care for people with dementia living in nursing homes. Dementia..

[26] Koskenniemi J, Leino-Kilpi H, Suhonen R (2015). Manifestation of respect in the care of older patients in longterm care settings. Scand J Caring Sci.

[27] Edvardsson D, Sjögren K, Lood Q, Bergland Å, Kirkevold M, Sandman P-O (2017). A person-centred and thriving-promoting intervention in nursing homes - study protocol for the U-age nursing home multi-Centre, non-equivalent controlled group before-after trial. BMC Geriatr.

[28] Vossius C, Selbæk G, Šaltytė Benth J, Bergh S (2018). Mortality in nursing home residents: A longitudinal study over three years. PLoS One.

[29] Verho H, Arnetz J (2003). Validation and application of an instrument for measuring patient relatives’ perception of quality of geriatric care. Int J Qual Health Care.

[30] Lindahl J, Elmqvist C, Thulesius H, Edvardsson D (2015). Psychometric evaluation of the Swedish language person-centred climate questionnaire–family version. Scand J Caring Sci.

[31] Pallant J (2010). SPSS survival manual: a step by step guide to data anlysis using SPSS.

[32] Shrive F, Stuart H, Quan H, Ghali W (2006). Dealing with missing data in a multi-question depression scale: a comparison of imputation methods. BMC Med Res Methodol.

[33] Edvardsson D, Sandman P, Rasmussen B (2008). Swedish language person-centred climate questionnaire - patient version: construction and psychometric evaluation. J Adv Nurs.

[34] Jakobsen R, Sellevold G, Egede-Nissen V, Sørlie V (2019). Ethics and quality care in nursing homes: relatives’ experiences. Nurs Ethics.

[35] Wiig S, Storm M, Aase K, Gjestsen M, Solheim M, Harthug S (2013). Investigating the use of patient involvement and patient experience in quality improvement in Norway: rhetoric or reality?. BMC Health Serv Res.

[36] Chenoweth L, Forbes I, Fleming R, King MT, Stein-Parbury J, Luscombe G (2014). PerCEN: a cluster randomized controlled trial of person-centered residential care and environment for people with dementia. Int Psychogeriatr.

[37] Rosher RB, Robinson S (2005). Impact of the Eden alternative on family satisfaction. J Am Med Dir Assoc.

[38] Kaplan H, Brady P, Dritz M, Hooper D, Linam W, Froehle C (2010). The influence of context on quality improvement success in health care: a systematic review of the literature. The Milbank Quarterly.

